# PARN deadenylase is involved in miRNA-dependent degradation of TP53 mRNA in mammalian cells

**DOI:** 10.1093/nar/gkv959

**Published:** 2015-09-22

**Authors:** Xiaokan Zhang, Emral Devany, Michael R. Murphy, Galina Glazman, Mirjana Persaud, Frida E. Kleiman

**Affiliations:** 1 Chemistry Department, Belfer Research Building, Hunter College and Graduate Center, City University of New York, New York, NY 10021, USA; 2 Department of Biological Sciences, Kingsborough Community College, City University of New York, 2001 Oriental Boulevard, Brooklyn, NY 11235, USA

## Abstract

mRNA deadenylation is under the control of cis-acting regulatory elements, which include AU-rich elements (AREs) and microRNA (miRNA) targeting sites, within the 3′ untranslated region (3′ UTRs) of eukaryotic mRNAs. Deadenylases promote miRNA-induced mRNA decay through their interaction with miRNA-induced silencing complex (miRISC). However, the role of poly(A) specific ribonuclease (PARN) deadenylase in miRNA-dependent mRNA degradation has not been elucidated. Here, we present evidence that not only ARE- but also miRNA-mediated pathways are involved in PARN-mediated regulation of the steady state levels of TP53 mRNA, which encodes the tumor suppressor p53. Supporting this, Argonaute-2 (Ago-2), the core component of miRISC, can coexist in complexes with PARN resulting in the activation of its deadenylase activity. PARN regulates TP53 mRNA stability through not only an ARE but also an adjacent miR-504/miR-125b-targeting site in the 3′ UTR. More importantly, we found that miR-125b-loaded miRISC contributes to the specific recruitment of PARN to TP53 mRNA, and that can be reverted by the ARE-binding protein HuR. Together, our studies provide new insights into the role of PARN in miRNA-dependent control of mRNA decay and into the mechanisms behind the regulation of p53 expression.

## INTRODUCTION

Modulation of the length of poly(A) tail of an mRNA by the polyadenylation/deadenylation machinery is a widespread strategy used to control mRNA stability and gene expression in different cellular conditions, such as development, mRNA surveillance, inflammatory response, cell differentiation, cancer and the DNA damage response (DDR) (1–3). The dynamic nature of the mRNA 3′-end processing machinery allows the regulation of the steady-state levels of different mRNAs and has the potential to contribute to the cells rapid response to stress. Poly(A) specific ribonuclease (PARN), a poly(A) specific 3′ exoribonuclease, has been shown to play a role in DDR (4,5). The association of nuclear PARN with the cleavage stimulation factor 50 (CstF-50) inhibits mRNA 3′ cleavage and activates deadenylation in the nucleus upon UV-induced DNA damage (4). Besides, PARN is also activated by tumor suppressors and DNA repair factors with compromised expression on most cancers, such as BARD1/BRCA1 (4) and p53 (5). Interestingly, PARN expression is altered in different cancers (4,6). PARN can regulate the stability of mRNAs of genes involved in DDR, such as c-myc, c-fos, c-jun and transcripts in the p53 and BARD1/BRCA1 pathways, keeping their levels low under non-stress conditions (4,5,7).

Deadenylation, and consequently mRNA stability, is under the control of cis-acting regulatory elements (1–3). Some of those signals are present in the 3′ untranslated region (3′ UTRs) of eukaryotic mRNAs, such as AU-rich elements (AREs) and microRNA (miRNA) targeting sites. In the last years, many studies have focused on the physiological relevance of the functional connection between these cis-acting elements and the 3′ processing machinery (8–13). Although some studies have shown that ARE-mediated decay can occur independent of miRNA functions (14), an increasing number of publications have shown that elements of the miRNA-induced silencing complex (miRISC) can functionally interact with ARE-binding proteins (BPs) (1). Although PARN is known to be involved in ARE-mediated deadenylation (15–17), the functional interaction of PARN and the miRISC has not been elucidated. Numerous studies have shown that other deadenylation complexes, such as CAF1/CCR4/NOT1 and Pan2–Pan3, are involved in miRNA-mediated deadenylation resulting in the regulation of mRNA stability and gene expression (reviewed in (1). The CCR4–NOT complex is the predominant deadenylase in all biological systems and for most mRNAs examined (18,19). Deadenylation and mRNA decay efficiency differ between mRNAs as the CCR4–NOT complex is recruited to specific mRNAs by means of either sequence-specific RNA-BPs or miRNAs.

In previous studies, we have shown that PARN affects half-life and poly(A) tail length of TP53 mRNA under non-stress conditions through an ARE present in the 3′ UTR (5). In this study, we found that PARN deadenylase regulates TP53 mRNA stability through not only an ARE but also an adjacent miR-504/miR-125b-targeting site in the 3′ UTR. Interestingly, the binding of PARN to the TP53 mRNA 3′ UTR depends on both cis-acting signals present in this region and Ago-2 expression. Besides we found that Ago-2 activates PARN deadenylase activity by directly interacting with the N-terminal domain of PARN and forming a complex. We also showed that the miR-125b-loaded miRISC can recruit PARN to the target TP53 mRNA, and this can be reverted by the ARE-BP HuR. This is the first report to show that PARN plays a role in regulating mRNA processing in a miRNA-dependent pathway in mammalian cells. This study reveals a regulatory pathway wherein a functional interplay of PARN deadenylase, RNA-BPs and elements of the miRISC is important to regulate the steady-state levels of TP53 mRNA and the progression of DNA damage response.

## MATERIALS AND METHODS

### Tissue culture methods and DNA damaging agents

HCT116 cell line was cultured and UV-treated as described (5,20,21).

### Nuclear Extracts (NEs) preparation

After UV treatment, NEs were prepared from harvested cells as described (4,20). The NEs were quickly frozen and stored at −80°C.

### Deadenylation with ^32^P-labeled polyadenylated RNA encompassing TP53 3′ UTR

WT and mutant p53 3′ UTRs were amplified from luciferase constructs by polymerase chain reaction (PCR) using a forward primer including a T3 promoter and a reverse primer with 20 adenines to create poly(A) tail at the 3′ end of the transcript (Forward 5′-ATGGATTCAATTAACCCTCACTAAAGGGAACATTCTCCACTTCTTGTTCCCCACTAC-3′ and Reverse 5′-GGATGATCCATAAGCTT(A)_20_TGG GATATAAAAAGGG-3′). The PCR fragments were digested with Hind III to generate the poly(A) tail. Then polyadenylated radiolabeled RNA substrates were synthesized by *in vitro* transcription with T3 polymerase as described (4,5) and used in deadenylation reaction with NEs as indicated in the figure.

### Deadenylation assays

^32^P-labeled L3(A_30_) substrates were prepared and analyzed as in (4,5). Deadenylation assays using equivalent amounts of total proteins from NEs from different cells were performed at 30°C for the incubation times indicated in each figure. Protein concentrations of the extracts were equalized by Bradford assays (Bio-Rad) before used in deadenylation reactions.

### Purification of recombinant proteins

A plasmid encoding the full-length Ago-2 was transformed into Rosetta cells and GST-fusion protein was purified by binding to and elution from glutathione–agarose beads as described in (22). Recombinant His-PARN and its derivatives were purified as described in (23).

### Pull-down assays

Interaction assays using His-PARN and GST-Ago-2 were performed as described (24). Equivalent amounts of pellets and supernatants were analyzed by immunoblotting.

### Immunoprecipitation (IP) analysis

100 μg total protein from the indicated NEs were immunoprecipitated (IPed) with polyclonal antibody against either PARN (H-105, Santa Cruz Biotechnology) or Ago-2 as described (4,20).

### siRNA-mediated knockdown of deadenylases

siRNAs specific for PARN (D-011348–04), Ago-2 (D-004639–01), Pan2 (D-021192–01), CCR4a (D-019101–01), HuR and control RNA duplexes were synthesized by Dharmacon RNA technologies (Lafayette, CO, USA). siRNA and UV-treatments (40 J/m^2^) were as described (4,20). Knockdown of proteins was confirmed by western blot analysis using following antibodies: Ago-2 (H-300, Santa Cruz), PARN (kindly provided by Dr A. Virtanen), Topo II (H-8, Santa Cruz), Pan2 (USP52, Santa Cruz). Knockdown of CCR4 was confirmed by quantitative PCR (qPCR) using following primers: Forward: 5′ GAAATGCCGTCTGGAAAGCC-3′ and Reverse: 5′-CCAATGCATGTGGGCGTTAG-3′. Rescue assays were performed by co-transfecting HCT116 cells with either siRNA-targeting PARN or control siRNA and/or WT/D28A PARN expression plasmids. PARN expression constructs were generated by subcloning the full-length coding sequence of PARN into the p3xFLAG pCMV10 expression vector (Sigma). Ala substitution for Asp 28 was introduced with the QuikChange Lightning Site-Directed Mutagenesis Kit (Agilent Technologies).

### Cellular fractionation assays

The fractionation of HCT116 cells was performed using Subcellular Protein Fractionation kit (Thermo Scientific) following the manufacturer's protocol. Equivalent amounts of cytoplasmic and nuclear component were subjected to SDS-PAGE and proteins were detected by immunoblotting using antibodies against Ago-2, PARN, Topo II and actin.

### Ago-2-siRNA knockdown and miRNA inhibitor expression plasmid transfection

siRNAs specific for human Ago-2 and control RNA duplex were synthesized by Dharmacon RNA technologies (Lafayette, CO, USA). siRNA - and UV-treatments (40 J/m^2^) were as described (4,20). Either miR-125b inhibitor expression plasmid (HmiR-AN0096-AM03, GeneCopoeia) or control plasmid (AM03, GeneCopoeia) were transfected into HCT116 cells using Lipofectamine TM 2000 reagent (Invitrogen).

### Constructs of luciferase reporter vectors

Luciferase vector pEZX-MT01 with TP53 miTarget™ miRNA 3′ UTR target clones (product ID: HmiT054283) was purchased from GeneCopoeiaTM. Mutations in the miRNA targeting sites, ARE sequence or both signals of p53 3′ UTR were introduced with the QuikChange Lightning Site-Directed Mutagenesis Kit (Agilent Technologies) and the following primers 5′-GGGTCAATTTCCGTTCGCGAATTCTGTTCTGATCTGCTTTTTCTTTGAGACTGGG -3′ and 5′-CCCAGTCTCAAAGAAAAAGCAGATCAGAACAGAATTCGCGA-ACGGAAATTGACCC-3′ for ARE sequence replacement, primers 5′- CTGGA-TCCACCAAGACTTGTTTTATGATTTCTTTTTTCTTTTTT-3′ and 5′-AAAAAAGA-AAAAAGAAATCATAAAACAAGTCTTGGTGGATCCAG-3′ for miRNA targeting sites replacement and primers 5′-CCAAGACTTGTTTTATGCATGTCCGTTCGCG-AATTCTGCTGTGATCTGCTTTTTCTTTGAGACTGG -3′ and 5′-CCAGTCTCA-AAGAAAAAGCAGATCACAGCAGAATTCGCGAACGGACATGCATAAAACAAGTCTTGG -3′ for both signals replacement following the manufacturer's instructions. Plasmids were sequenced to confirm the presence of the mutation. 24 μg of the different luciferase constructs were transfected into cells using Lipofectamine^TM^ 2000 reagent (Invitrogen).

### Luciferase assay

Cells were co-transfected with 24 μg luciferase constructs (Lipofectamine TM 2000 reagent, Invitrogen) and either siRNA-targeting PARN or control siRNA. 48 h after transfection cells were harvested and dual luciferase assay was performed using Luc-pair miR Luciferase kit from GeneCopoeia (Rockville, MD, USA) following manufacturer's instructions.

### RNA IP (RIP) assays

IP of nuclear RNA–protein complexes was performed as described (25). Briefly, cells were treated with 1% formaldehyde and NEs were then prepared followed by sonication. Extracts were treated with DNase (TURBO DNA-free Kit, Ambion), and the resulting material was IPed with antibodies against PARN or control rabbit IgG (Sigma). Protein–RNA complexes were treated with proteinase K and reversal of cross-linking. RNA was extracted from the IPs with phenol-chloroform and analyzed by quantitative reverse transcription PCR (RT-qPCR) assays.

### Ribonuclease protection assays (RPA)

RIP was performed using either PARN, Ago-2 or IgG antibodies; NEs from HCT116 cells; and a radiolabeled RNA derivative of WT TP53 3′ UTR. Samples were crosslinked before RIP. After RIP, samples were treated with micrococcal nuclease (MNase, Life Technologies). Then, the protein fraction was eliminated by proteinase K treatment, phenol/chloroform extraction and precipitation. Samples were then analyzed in denaturing urea gels followed by autoradiography.

### RT-qPCR assays

As described before (4,20), equivalent amounts (2 μg) of purified RNA were used as a template to synthesize cDNA using random hexamer primers, oligo-d(T)primers and GoScript Reverse Transcriptase (Promega). Relative levels were calculated using ΔCτ method.

### RNA pull-down

Biotin-labeled RNAs were in vitro transcribed with the biotin RNA labeling mix (Roche), T3 RNA polymerase (Promega) following manufacturer's instructions. The primers used to generate these fragments were: forward 5′-ATGGATTCAATTAACCCTCACTAAAGGGAAGGGCAGCTGGTTAGGTAGAG-3′ and reverse 5′- GGAATAAGCTT(A)_20_CACCCCTCAGACACACAGG-3′ (648 nt); and forward 5′ AGCCGCATTAACCCTCACTAAAGGGATCTCTTGTATATGATG-3′ and 5′-GGAATAAGCTT(A)_20_CCTGGGCAACAAAGCGA-3′ (123 nt). Folded RNA was then mixed with 1 mg of either NEs or cytoplasmic fractions and analyzed as described before (4,20).

### RACE-poly(A) test assays

Nuclear RNA from HCT116 cells treated with Ago-2/control siRNA was reverse-transcribed using oligo (dT)-anchor primer and GoScript Reverse Transcriptase (Promega). Poly(A) tail length of p53 mRNA was analyzed as described before (5).

### RNA isolation and RT-qPCR analysis of miRNA abundance

Nuclear RNA and total RNA were purified using RNeasy Mini Kit (QIAGEN) according to the manufacturer's directions. miRNAs abundance was assessed by RT-qPCR using All-in-One™ miRNA RT-qPCR Reagent Kits and Validated Primers (GeneCopoeia).

### Immunoflourescence and confocal microscopy

HCT116 cells were seeded onto glass coverslips at 5 × 104 cells/ml, immunostained for PARN (H-105, Santa Cruz Biotechnology) and Ago-2 (H-300, Santa Cruz) as described (26). Cells were examined under a Nikon TIRF SIM laser spinning disk fluorescence confocal microscope.

## RESULTS

### PARN regulates p53 expression through not only ARE but also miRNA targeting site present in the 3′ UTR of p53 mRNA

**Figure 1.**
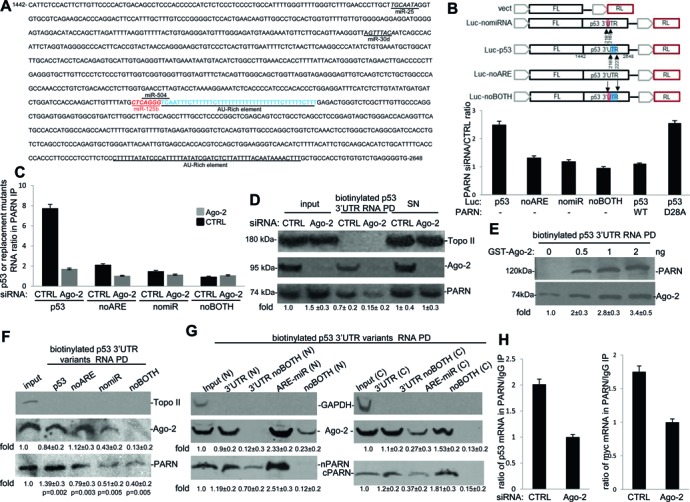
Ago-2 and adjacent ARE-miRNA targeting sites present in the 3′ UTR of TP53 mRNA are required for PARN recruitment. **(A)** Sequence of TP53 mRNA 3′ UTR. miRNA targeting sites and AU-rich elements (AREs) are shown. miR-125b/miR-504 binding site (red) are adjacent to an ARE (blue). **(B)** Both AREs and adjacent miRNA targeting sites are critical for PARN-mediated regulation of p53 expression. Diagram of firefly luciferase reporter constructs with different 3′ UTR sequences from the TP53 gene. The sites of replacement are indicated: miR-125b/miR-504 binding site (red), ARE (blue). Constructs with different 3′ UTR sequences from the p53 gene were transfected into HCT116 cells treated with control or PARN siRNA. The ratio of the firefly/Renilla values obtained for each construct in PARN knockdown cells relative to control siRNA-treated cells are shown. Expression of WT PARN, but not the catalytically inactive D28A PARN, can revert the increase in the ratio of firefly/renilla luciferase activity observed after siRNA-mediated knockdown of PARN. The firefly/renilla values were calculated from three independent samples. Errors represent the SD derived from three independent experiments. **(C)** Ago-2 expression and overlapping ARE/miRNA targeting signals in TP53 3′ UTR are involved in PARN association 3′ UTR TP53. RIP assays were performed using samples from HCT116 cells treated with Ago-2/control siRNAs and transfected with the luciferase constructs described in (B). NEs were IPed with either antibodies against PARN or control IgG. Nuclear RNA IPed was quantified by qRT-PCR using primers specific for luciferase gene. Errors represent the SD (*n* = 3). **(D)** PARN interacts with the TP53 3′ UTR in an Ago-2-dependent manner. RNA pull-down assays were performed using biotinylated RNA carrying part of WT TP53 3′ UTR sequence and NEs from HCT116 cells treated with Ago-2 or control siRNAs. A representative pull-down reaction from three independent assays is shown. Ten percent of the NE used in the pull-down reactions is shown as input. The means ± standard deviation of PARN signals are indicated. **(E)** Recombinant Ago-2 facilitates PARN binding to part of TP53 mRNA 3′ UTR. *In vitro* RNA pull-down assays were carried out using NEs from HCT116 cells treated with Ago-2 siRNA, biotinylated RNA encompassing the WT TP53 3′ UTR and increasing amount of recombinant GST-Ago-2. A representative reaction from three independent assays is shown. The means ± standard deviation of PARN signals are indicated. **(F** and **G)** Both ARE and adjacent miR-125b targeting signal at TP53 3′ UTR are necessary for PARN binding. RNA pull-down assays were performed using biotinylated RNA carrying WT or signal replaced (noARE, nomiR and noBOTH) TP53 3′ UTR and NEs from HCT116 cells. A shorter RNA carrying only the ARE adjacent to miR-125b targeting signal was analyzed in (G). Nuclear and cytoplasmic fractions were analyzed in (G). A representative pull-down reaction from three independent assays is shown. Ten percent of the NE used in the pull-down reactions is shown as input. The means ± standard deviation of PARN and Ago-2 signals are indicated. **(H)** Ago-2 is required for the association of PARN to its target mRNAs under non-stress conditions. NEs from HCT116 cells treated with Ago-2/control siRNA were prepared after formaldehyde crosslinking. RIP assays were performed as before in (C). The endogenous nuclear RNA IPed was quantified by qRT-PCR using primers specific for TP53 and c-myc genes. The PARN/IgG ratios were further normalized, making arbitrarily the ratio for Ago-2 knockdown samples 1. The qRT-PCR values were calculated from three independent samples. Errors represent the SD (*n* = 3).

PARN regulates the stability of genes involved in DDR, such as TP53 and c-myc, by indirect association to their transcripts keeping their levels low under non-stress conditions (4,5,7). PARN depletion increases the half-life of the TP53 transcript under non-stress conditions due to an ARE present in the 3′ UTR (5). AREs may consist of the AUUUA element in various arrangements or regions of Us only (27). The 3′ UTR of p53 harbors one U-rich region (18 continuous Us) and one additional ARE containing the AUUUA motif (2,5,28,29). PARN-mediated regulation of TP53 mRNA is lost by the replacement of the ARE with the U-rich region (encompassing positions 2181 to 2223). Interestingly, miR-504 (30) and miR-125b (31) targeting sites are physically adjacent to this ARE (28,29) in TP53 3′ UTR (Figure 1A). While PARN is known to be involved in ARE-mediated deadenylation (15–17), its role in miRNA-mediated deadenylation has not been elucidated. To test whether miRNA-targeting sites adjacent to an ARE were involved in PARN-mediated regulation of TP53 mRNA steady state levels we used constructs that have the luciferase gene carrying either WT or replacement mutants in AREs and/or miR-504/miR-125b-targeting sequences in the TP53 3′ UTR (Figure 1B). As miR-504 and miR-125b have similar targeting sequences, the miRNA replacement mutant designed affected the recognition of both miRNAs. The miR-25 and miR-30d targeting sequences, shown in Figure 1A, were not modified in those constructs. Interestingly, the siRNA-mediated knockdown of PARN significantly increased the ratio of firefly/renilla luciferase activity from the constructs carrying the WT TP53 3′ UTR, and this was abolished when the AREs (noARE), miR-504/miR-125b target sites (nomiR) or both (noBOTH) signals were replaced by other sequences (Figure 1B). As the miR-25 and miR-30d targeting sequences were present in those replacement mutants, we inferred that the presence of those targeting sites was not sufficient to rescue PARN-mediated regulation. Importantly, expression of WT PARN, but not the catalytically inactive D28A PARN (32), can revert the increase in luciferase activity observed with constructs carrying the WT TP53 3′ UTR after siRNA-mediated knockdown of PARN (Figure 1B). The expression levels of both WT and catalytically inactive PARN was similar (Supplementary Figure S1A), indicating that the observed effect on the luciferase reporters was due to catalytic activity of PARN. These rescue experiments indicate that the effects observed with the TP53 reporter constructs are specific for PARN and its deadenylase activity.

Next, we examined whether PARN physically associates with TP53 mRNA through ARE and/or adjacent miRNA targeting sites in the 3′ UTR. It is important to highlight that since PARN is primarily localized to the nucleus (33–35) and our previous work has focused only in nuclear functions of PARN (4,5,20), here we have continued studying PARN functions in nuclear fractions. Our RIP assays with cross-linked nuclear RNA indicated that PARN can form a complex with the luciferase mRNA carrying the WT 3′ UTR of TP53 but not with the replacement mutants in AREs and/or miRNA-targeting sequences in the TP53 3′ UTR (Figure 1C, black bars). The presence of miRNA targeting sequences that were not adjacent to the ARE, such as miR-25 and miR-30d, in the luciferase mRNA carrying the replacement mutant 3′ UTR of TP53 was not sufficient for PARN binding to the RNA (Figure 1C). Together, these results indicate that both ARE and the adjacent miR-504/miR-125b regulatory signals at the TP53 3′ UTR are necessary for PARN-mediated regulation of p53 expression.

### Association of PARN to its target mRNAs requires Ago-2

To further analyze the role of miRNA targeting sites in PARN-mediated regulation of p53 mRNA stability we tested whether Ago-2, which is one of the core components of the miRISC complex that delivers miRNAs and deadenylases to their mRNA targets, is involved in the recruitment of PARN to its mRNA targets. We decided to start our study with Ago-2 because a functional interaction between PARN and Ago-2 and their role in miRNA biogenesis pathway were recently described (36). Using the luciferase constructs described in Figure 1B, we determined that the interaction of PARN with the luciferase mRNA carrying the WT 3′ UTR of TP53 was lost in HCT116 cells treated with Ago-2 siRNA (Figure 1C, gray bars). Interestingly, Ago-2 knockdown and the replacement of the ARE and/or adjacent miR-504/miR-125b regulatory signals in TP53 3′ UTR had a similar effect on PARN-luciferase mRNA interaction. Together, these results support the idea that PARN functionally interacts with not only ARE but also elements of the miRISC in adjacent targeting sites.

To rule out the possibility that the effect of depletion of Ago-2 on reducing PARN association with TP53 mRNA 3′ UTR is due to the cell-wide response to low levels of Ago-2 expression, RNA pull-down assays were performed using an *in vitro* transcribed biotinylated RNA encompassing WT TP53 3′ UTR, NEs from HCT116 cells treated with Ago-2 or control siRNAs. Our results indicate that a biotinylated RNA encompassing WT TP53 3′ UTR pulled-down PARN from samples of control siRNA-treated cells, and this RNA–PARN interaction significantly decreased when samples from Ago-2 siRNA-treated cells were used in the assay (Figure 1D). Addition of increasing amounts of recombinant Ago-2 to NEs samples from cells depleted in Ago-2 increased the amounts of PARN detected in the WT TP53 3′ UTR pull-down fraction (Figure 1E). Moreover, RNA pull-down assays using biotinylated RNAs encompassing either WT or mutant variants of TP53 3′ UTRs, and NEs from HCT116 cells showed that the RNA–PARN interaction depended on the presence of both the ARE sequence and the adjacent miR-504/miR-125b targeting site (Figure 1F). As expected, the interaction of Ago-2 with the RNA was not affected by the replacement of the ARE. As the biotinylated RNAs did not encompass miRNA targeting sequences that were not adjacent to the ARE, the binding of Ago-2 to miR-25 and miR-30d targeting sequences was not detected in these assays. Interestingly, while the loss of the miR-504/miR-125b targeting site decreased Ago-2 binding to the biotinylated RNAs, the loss of the ARE using the noBOTH reporter further reduces Ago2 binding. Consistent with this, our results in Figure 4E indicate that the ARE-BP HuR affects the binding of Ago-2 to TP53 mRNA, supporting the idea that the association of an ARE-BP may affect Ago-2 biding to TP53 3′ UTR. Similar functional interaction between ARE-BP and miRISC has been described by others (37,38). Some PARN was pulled-down by the noBOTH construct, probably due to the presence of the most distal ARE of TP53 3′ UTR in the biotinylated RNAs. Consistent with this, in Figure 1D and E, we showed that PARN can bind biotinylated WT p53 RNA in the absence of Ago-2, indicating that other signal(s) present in those RNAs might be recruiting PARN.

Importantly, both PARN and Ago-2 were pulled-down by a shorter RNA encompassing only the ARE adjacent to the miR-504/miR-125b-targeting site of the TP53 3′ UTR (Figure 1G). The interaction was lost in the noBOTH derivative of this fragment. These results indicate that the presence of ARE adjacent to the miR targeting site is sufficient for the interaction of PARN and Ago-2 with TP53 3′ UTR. The PARN/Ago-2 interaction with TP53 3′ UTR was also analyzed by RPA (Supplementary Figure S1D). We performed RNA-IP using either PARN or Ago-2 antibodies, NEs from HCT116 cells and a radiolabeled RNA encompassing the ARE adjacent to the miR-504/miR-125b-targeting site of the TP53 3′ UTR (123 nt) described above. Protein fractions were eliminated from the samples after MNase treatment. These protection assays showed a fragment of a similar size (75 nt) from RIP samples using either PARN or Ago-2 antibodies, indicating that PARN and Ago-2 bind the same region of TP53 3′ UTR as part of the same complex.

Then we determined whether a similar association between PARN, Ago-2 and the TP53 mRNA persist in the cytoplasm. Previous studies showed that PARN (39,40) can localize in both nuclear and cytoplasmic fractions. Consistent with this, our results indicate that an RNA encompassing only the ARE adjacent to the miR-504/miR-125b-targeting site of the TP53 3′ UTR was able to pull-down PARN and Ago-2 from both cellular fractions (Figure 1G). Together, these results, and the localization studies shown in Figure 3C and D, suggest that PARN-mediated deadenylation TP53 mRNA might be a phenomenon that can occur in both nuclear and cytoplasmic compartments. Consistent with this, the results shown in Figure 3B indicate that the N-terminal domain of PARN interacts directly with Ago-2. As the differences between nuclear and cytoplasmic PARN at the N-terminus are minimal, the interaction of PARN/Ago-2/TP53 3′ UTR RNA in both cellular compartments is not surprising. Interestingly, p53 protein levels increased in samples from cells treated with either Ago-2 or PARN siRNA (Supplementary Figure S1B), supporting the idea that both factors might be involved in the same p53 regulatory pathway (5). In fact, the double knockdown of PARN and Ago-2 resulted in similar p53 protein levels to Ago-2 knockdown alone indicating that Ago-2 is necessary for PARN-mediated reduction of p53 protein levels. Extending these studies, we examined whether Ago-2 expression is necessary for PARN association with its mRNA targets using nuclear samples. RIP assays were performed with antibodies against PARN and NEs from HCT116 cells treated with either control or Ago-2 siRNA. Consistent with our previous report (5), our RIP assays showed that both p53 and c-myc mRNAs formed a complex with PARN in cross-linked nuclear samples (Figure 1H). Interestingly, siRNA-mediated knockdown of Ago-2 reduced by approximately half the previously described interaction of PARN with both p53 and myc mRNAs (5). Together, these results indicate that Ago-2 plays a critical role in PARN-mediated regulation of gene expression by recruiting PARN to its target mRNAs.

### PARN functionally interacts with miR-125b-associated Ago-2

**Figure 2.**
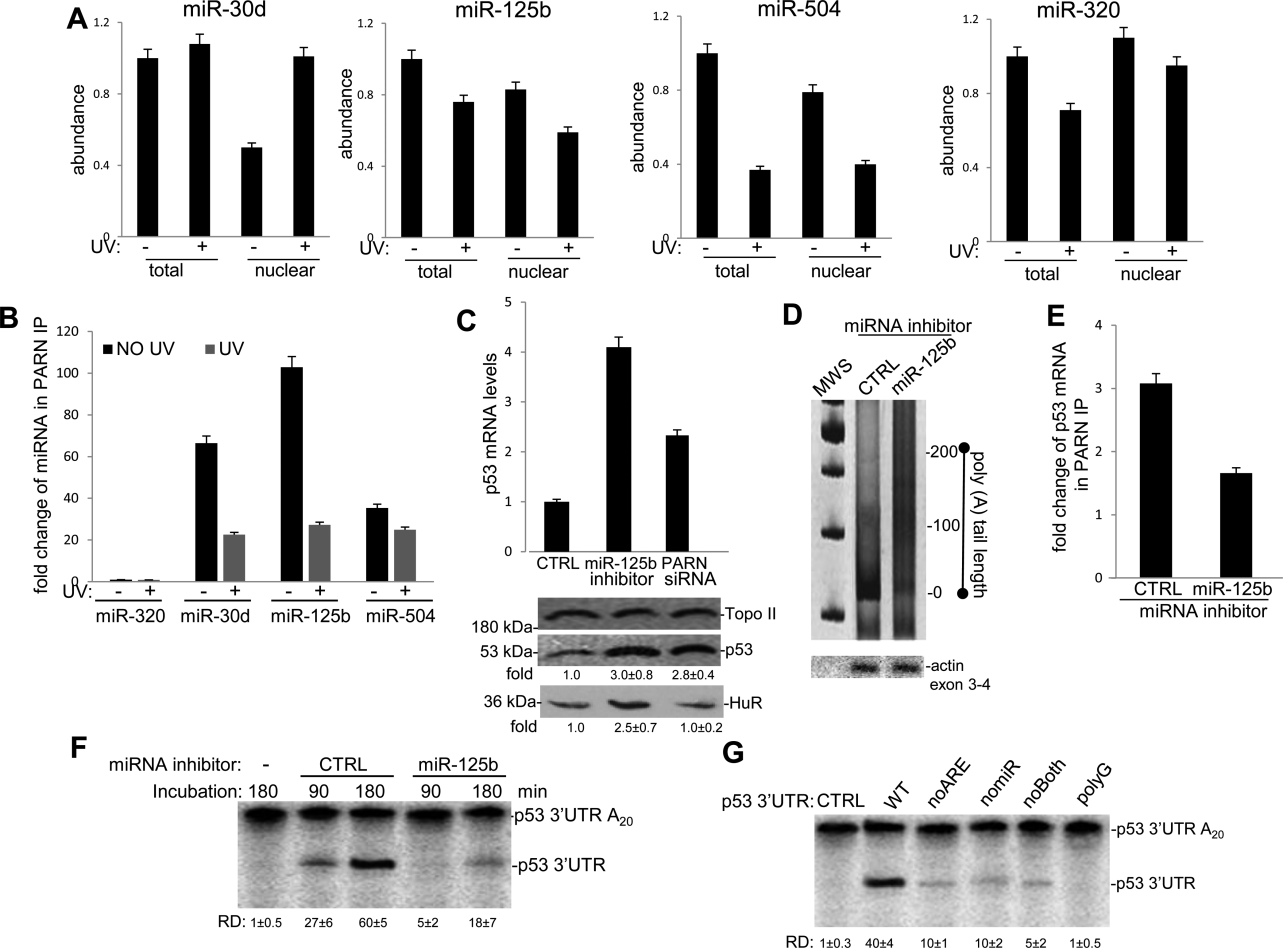
miR-125b contributes to the recruitment of PARN to target TP53 mRNA. **(A)** Expression profile of TP53 targeting miRNAs in response to UV irradiation. Nuclear and total RNAs from UV treated HCT116 cells were isolated and miRNA abundance was assessed by qRT-PCR using a specific kit for miRNA analysis and normalized to actin. Errors represent the SD derived from three independent experiments. **(B)** PARN association to different TP53 targeting miRNAs is favored in non-stress conditions. RIP assays were performed as in Figure 1C. Nuclear miRNAs IPed were quantified by qRT-PCR. Errors represent the SD derived from three independent experiments. **(C)** Both functional knockdown of miR-125b and PARN increase TP53 mRNA and protein levels. Samples from HCT116 cells transfected with miR-125b inhibitor expression plasmids, control vectors or PARN siRNA were analyzed for TP53 mRNA levels by qRT-PCR, and for p53 and HuR protein levels by western blot. Errors represent the SD derived from three independent experiments. A representative western blot from three independent assays is shown. The means ± standard deviation of HuR and p53 signals are indicated. **(D)** miR-125b regulates p53 mRNA poly(A) tail length. Nuclear RNAs from HCT116 cells transfected with miR-125b inhibitor expression/control vectors were reverse-transcribed using an oligo(dT)-anchor primer and amplified using an oligonucleotide that hybridizes within the TP53′ UTR. The products were separated on a non-denaturing PAGE and detected by ethidium bromide staining. An RT-PCR product from a non-PARN target gene (ACTIN exon 3–4) was used as a loading control. A representative RACE-PAT analysis from three independent assays is shown. Molecular weight standard (MWS, 100 base pair ladder from Promega) is also included. **(E)** Functional loss of miR-125b attenuates TP53 mRNA association with PARN. RIP assays were performed as in (B), using NEs from HCT116 cells transfected with miR-125b inhibitor expression plasmids or control vectors. Errors represent the SD derived from three independent experiments. **(F)** Functional knockdown of miR-125b inhibits deadenylation of TP53 mRNA. Deadenylation assays were performed using NEs from HCT116 cells transfected with miR-125b inhibitor expression/control plasmids and a radiolabeled/polyadenylated RNA encompassing the WT TP53 3′ UTR. The incubation times for the deadenylation assay are indicated in the figure. Positions of the polyadenylated RNA (TP53 3′ UTR A_20_) and deadenylated product (TP53 3′ UTR) are indicated. A representative deadenylation reaction from three independent assays is shown. Numbers beneath gel lanes indicate relative deadenylation (RD). RD was calculated as [p53 3′ UTR/(p53 3′ UTR + p53 3′ UTR A_20_)] x 100. The means ± standard deviation of RD values are indicated. Quantifications were done with Image J software. **(G)** Regulatory signals at TP53 3′ UTR are important for deadenylation of TP53 mRNA. Deadenylation assays were performed using NEs from HCT116 cells and radiolabeled/polyadenylated (A_20_) RNA encompassing WT or signal replaced (noARE, nomiR and noBOTH) TP53 3′ UTRs. Deadenylation reactions were analyzed as in (F).

To further characterize the functional interaction of PARN with miRISC we first determined the changes in expression profiles during DDR and in different compartments of some of the miRNAs involved in p53 regulation. We analyzed miR-504, miR-125b and miR-30d, which have been previously shown to have a target site in TP53 3′ UTR and to downregulate p53 expression (30,31,41). We also included a miRNA that does not target TP53 mRNA (miR-320) in our assays. We determined the effect of UV-treatment on the abundance of these miRNAs in samples from HCT116 cells by qRT-PCR analysis of total and nuclear RNA samples. Consistent with previous reports (42,43), the total amount of each miRNA showed a change of different magnitude after UV treatment (Figure 2A). Changes in the abundance of these miRNAs were also observed in the nuclear RNA samples in response to DNA damage. As described before (44), our results also showed the enrichment of miR-320 in nuclear fractions. Interestingly, both total and nuclear levels of miR-125b and miR-504, whose targeting sequences are adjacent to an ARE in TP53 3′ UTR, decreased upon UV treatment. To examine whether PARN associates with these miRNAs, nuclear lysates from HCT116 cells were IPed with an antibody against PARN or control IgG followed by RT-qPCR detection of miRNAs. Interestingly, RIP assays indicate that under non-stress conditions miR-125b, miR-504 and miR-30d were enriched in PARN-IPed samples (Figure 2B). Our RIP assays did not show PARN association to miR-320. Remarkably, both miR-125b and miR-30d showed a decrease in PARN-association in response to UV-induced DNA damage, suggesting that PARN might have a critical role in miRNA-mediated downregulation of p53 expression under non-stress conditions. However, in Figure 1B and C, we showed that miRNA targeting sequences that were not adjacent to the ARE, such as the one for miR-30d, were not sufficient to bind and recruit PARN deadenylase to TP53 3′ UTR. Based on these results we speculate that the PARN-miR-30d complex might target other mRNAs than TP53. Interestingly, our preliminary search indicated that two PARN target genes have a miR-30d-targeting site adjacent to ARE sequence in their 3′ UTRs. Those genes are the connective tissue growth factor CTGF (45,46) and the transmembrane 4L6 family member 1 TM4SF1 (47,48). Further experiments are necessary to determine the functional relevance of a miR-30d-targeting site adjacent to ARE sequence on PARN-mediated regulation of mRNA stability.

To further analyze the role of miR-125b in PARN-mediated TP53 mRNA decay we transfected HCT116 cells with miR-125b inhibitor expression plasmid, which blocks endogenous miR-125b, or control plasmid and analyzed TP53 mRNA steady state levels. As miRNA inhibitors work as a ‘miRNA-sponges’ to form an entrapping structure and bind to their target miRNA, they prevent the binding of endogenous miRNA to target mRNA and inhibiting the effect of miRNA on target gene expression. Consistent with the studies of Le et al. (31), our results indicate that the functional knockdown of miR-125b increased TP53 mRNA (Figure 2C, upper panel) and protein levels (lower panel). Consistent with previous studies (30), the functional knockdown of miR-125b also resulted in an increase of HuR protein levels. Interestingly, as previously shown in our studies (5), PARN-knockdown also resulted in the increase of both TP53 mRNA and p53 expression with no effect on HuR protein levels (Figure 2C). Importantly, both PARN knockdown (5) and the functional inhibition of miR-125b resulted in the elongation of the poly(A) tail length of TP53 mRNA (Figure 2D and Supplementary Figure S2C). Extending these studies, we performed TP53 mRNA RIP assays using PARN antibodies and NEs from HCT116 cells transfected with miR-125b inhibitor expression or control plasmids. Interestingly, similar to the TP53 mRNA RIP assays with samples from Ago-2 depleted cells (Figure 1F), our RIP assays with samples from miR-125b depleted cells showed that functional inhibition of miR-125b decreased the binding of PARN to TP53 mRNA (Figure 2E).

To further characterize the regulatory function of miR-125b in TP53 mRNA deadenylation we used a cell free experimental system. Briefly, NEs from HCT116 cells transfected with miR-125b inhibitor or control vectors were analyzed for deadenylation assays using an *in vitro* transcribed, radiolabeled, capped and polyadenylated (A_20_) RNA encompassing the WT TP53 3′ UTR (Figure 2F and G) or the mutants shown in Figure 1B (Figure 2G). Importantly, samples from cells with functional loss of miR-125b showed a decrease in the deadenylation of WT TP53 3′ UTR (Figure 2F), indicating miR-125b is important to promote miRNA-mediated deadenylation of TP53 mRNA. In Figure 2G, we showed that the presence of not only ARE but also adjacent miR-504/miR-125b regulatory signals in TP53 3′ UTR were important for deadenylation of TP53. An RNA encompassing the WT TP53 3′ UTR with a poly(G_15_) was unaffected by addition of NEs from HCT116 cells to the deadenylation reaction (Figure 2G), indicating that the observed activity was a 3′ exonuclease and not endonucleolytic cleavage. Together, our results indicate that the recruitment of PARN to TP53 mRNAs requires regulatory signals in the 3′ UTR, ARE and adjacent miR-504/miR-125b signals, and miR-125b-loaded miRISC.

### The miRISC component Ago-2 binds and activates PARN deadenylase, and this can be reverted by HuR binding to ARE

**Figure 3.**
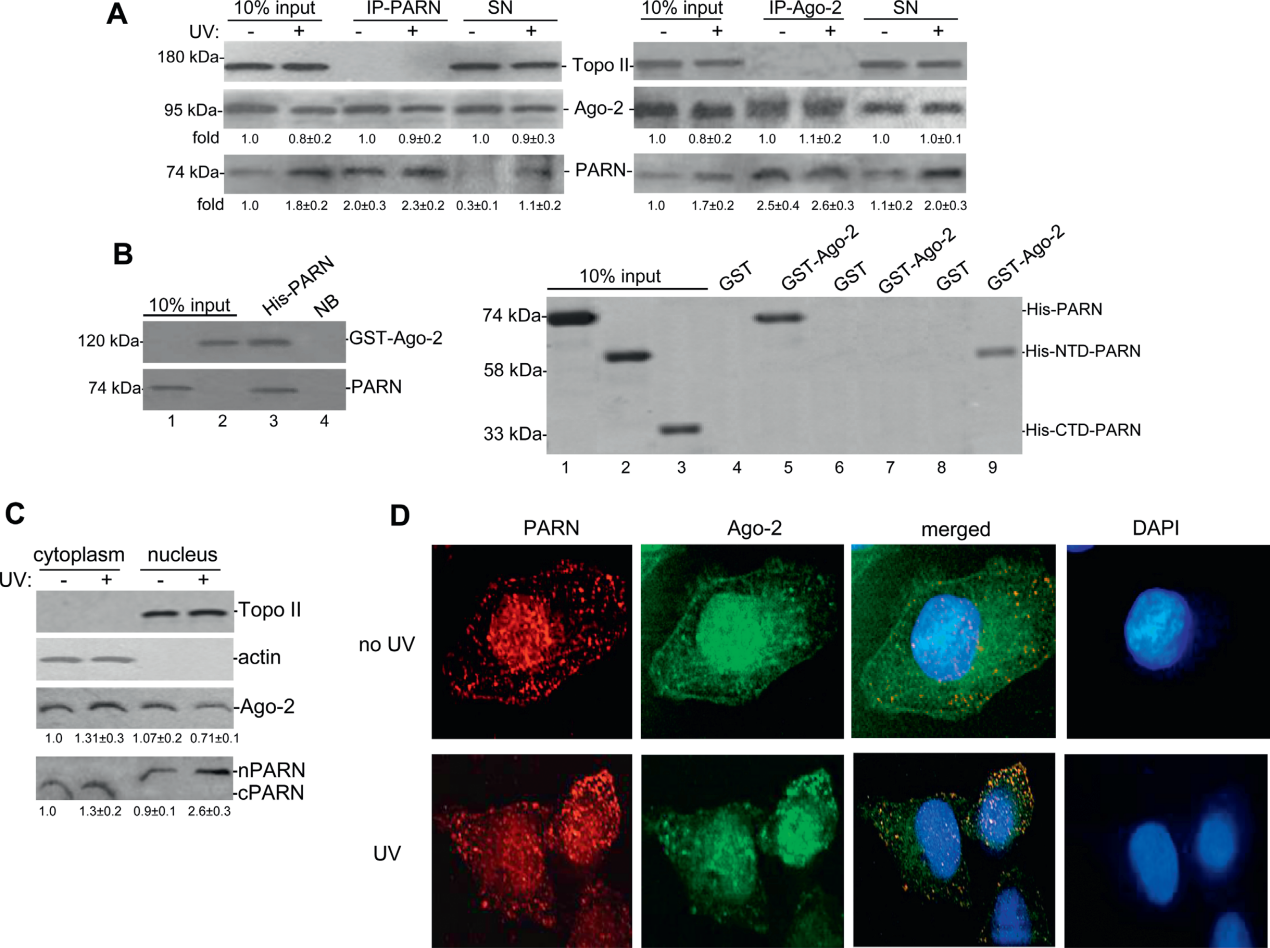
The miRISC component Ago-2 interacts with PARN deadenylase to form a protein complex. **(A)** PARN and Ago-2 could form (a) complex(es) in NEs of HCT116 cells independently of UV treatment. The NEs were IPed with anti-PARN (left) and anti-Ago-2 (right). Equivalent amounts of the pellets (IP) and supernatants (SN) were resolved by SDS-PAGE and proteins were detected by immunoblotting using antibodies against PARN and Ago-2. Antibody against Topo II was used as a control. A representative pull-down reaction from three independent assays is shown. The means ± standard deviation of PARN and Ago-2 signals are indicated. **(B)** Ago-2 interacts directly with the N-terminal domain of PARN. Immobilized His-PARN on nickel beads or nickel beads alones (NB) were incubated with GST-Ago-2 (left). Bound proteins were eluted, detected by western blotting with antibodies against PARN or Ago-2. 10% of either His-PARN or GST-Ago-2 used in the reaction are shown as input. Right panel: immobilized GST-Ago-2 or GST on glutathione beads were incubated with full-length (His-PARN), N-terminal domain (His-NTD-PARN; aa 1–470) or C-terminal domain (His-CTD-PARN; aa 443–639). A representative pull-down reaction from three independent assays is shown. Bound proteins were detected by immunoblotting as before. **(C** and **D)** Both PARN and Ago-2 are present in the nuclear and cytoplasmic fractions. Equivalent amounts of cytoplasmic and nuclear fractions of HCT116 cells were subjected to SDS-PAGE and proteins were detected by immunoblotting. Both nuclear (nPARN) and cytoplasmic (cPARN) isoforms of PARN are shown. Topo II and actin are used as subcellular fractionation control. The basal level of the proteins was arbitrarily set at 1.0 in the first lane, and relative fold change of each protein level is shown below each lane. A representative western blot from three independent assays is shown. Subcellular localization of PARN and Ago-2 was confirmed by confocal microscopy analysis. Representative thin sections of confocal fluorescence micrographs from HCT116 cells were fixed with 2% formaldehyde and immunostained with Ago-2 (monoclonal) and PARN (polyclonal) specific antibodies. Immunostaining with monoclonal and polyclonal antibodies were visualized with FITC conjugated anti-mouse and Cy5.5 conjugated anti-rabbit, respectively. Nucleus stained with DAPI is also shown.

To further understand the functional interaction between PARN deadenylase and components of the miRISC complex we tested the potential interaction of PARN deadenylase and Ago-2, the core component of the complex. To test whether PARN physically associates with Ago-2 we performed co-immunoprecipitation (co-IP, Figure 3A and Supplementary Figure S2D) and pull-down (Figure 3B, Supplementary Figure S2C) assays. The co-IPs indicate that PARN can form (a) protein complex(es) with Ago-2 in NEs from HCT116 cells. Interestingly, the complex was detected in both non-stress conditions and after UV treatment. As samples were treated with RNase A, the observed interactions were probably not due to an RNA tethering effect. Interestingly, PARN and Ago-2 interaction occurred without addition of miRNA to the pulled-down samples. The results showed that PARN can interact directly with Ago-2, and that PARN N-terminal domain is important for this interaction (Figure 3B, Supplementary Figure S2C), which includes the R3H domain (aa 135–268) and the catalytic nuclease domain (49). While the R3H domain can bind RNA with no oligo(A) specificity and plays a role keeping PARN dimeric structure, its removal does not affect RNA-binding ability and catalytic functions of PARN. PARN also possesses another RNA recognition motif RRM (aa 446–520), which play a critical role in PARN processivity. Although both R3H and RRM domains are essential for long poly(A) affinity, the R3H domain does not play a role in substrate recognition of PARN. Our results indicate that the complete RRM domain does not bind Ago-2. It is possible that the binding of Ago-2 to a region harboring the RH3 domain and the catalytic domain increases PARN affinity for different mRNAs and its deadenylase activity. Although a direct interaction between PARN and Ago-2 has not been previously described, recent studies have shown the functional interaction of PARN and Ago-2 during miRNA biogenesis (36). Together these results indicate that PARN can interact with the miRISC component Ago-2 to form (a) complex(es) independently of miRNA and stress conditions.

To further analyze the effect of UV treatment on expression levels and localization of both PARN and Ago-2 we performed western blot analysis of samples from different cellular fractions. We were able to distinguish the nuclear from the cytoplasmic forms of PARN because they differ in their molecular weight. While the 74 kDa isoform of PARN is exclusively nuclear, the 62 kDa isoform is cytoplasmic (39,40). Consistent with our previous studies (5) and as shown in Figure 1D, after UV treatment, we observed an increase in PARN levels in nuclear fractions (Figure 3C). However, we did not observed a significant change in PARN levels in cytoplasmic fractions. Subcellular localization studies in mammalian cells have shown that elements from the miRISC, such as Ago-1 and Ago-2, are localized both in the nucleus and cytoplasm (50–52). Consistent with this, our studies indicated that Ago-2 can be detected in both factions. Interestingly, a decrease in Ago-2 levels was observed in nuclear fractions after UV-treatment. We didn't detect a significant change of Ago-2 levels in cytoplasmic fractions after UV-treatment. These localization studies were further confirmed by confocal microscopy analysis (Figure 3D). Interestingly, our results indicated that both proteins localized in both compartments in either discrete foci or a more diffuse pattern. The foci for both proteins were more abundant in the cytoplasm than in the nucleus. Importantly, we observed overlapping of PARN and Ago-2 foci, supporting our biochemical studies that showed the direct interaction of PARN with Ago-2 (Figure 3A and B). Although Ago-2 seems to form more foci after UV-treatment, we did not observe a change in the co-localization of PARN and Ago-2 in those conditions. This is consistent with the co-IP analysis (Figure 3A) that showed that PARN can form a complex with Ago-2 before and after UV-treatment. Together, these studies indicate that PARN and Ago-2 can localize in both nuclear and cytoplasmic fractions, and as shown in Figure 1G, TP53 mRNA/PARN/Ago-2 complexes can be formed in both compartments.

**Figure 4.**
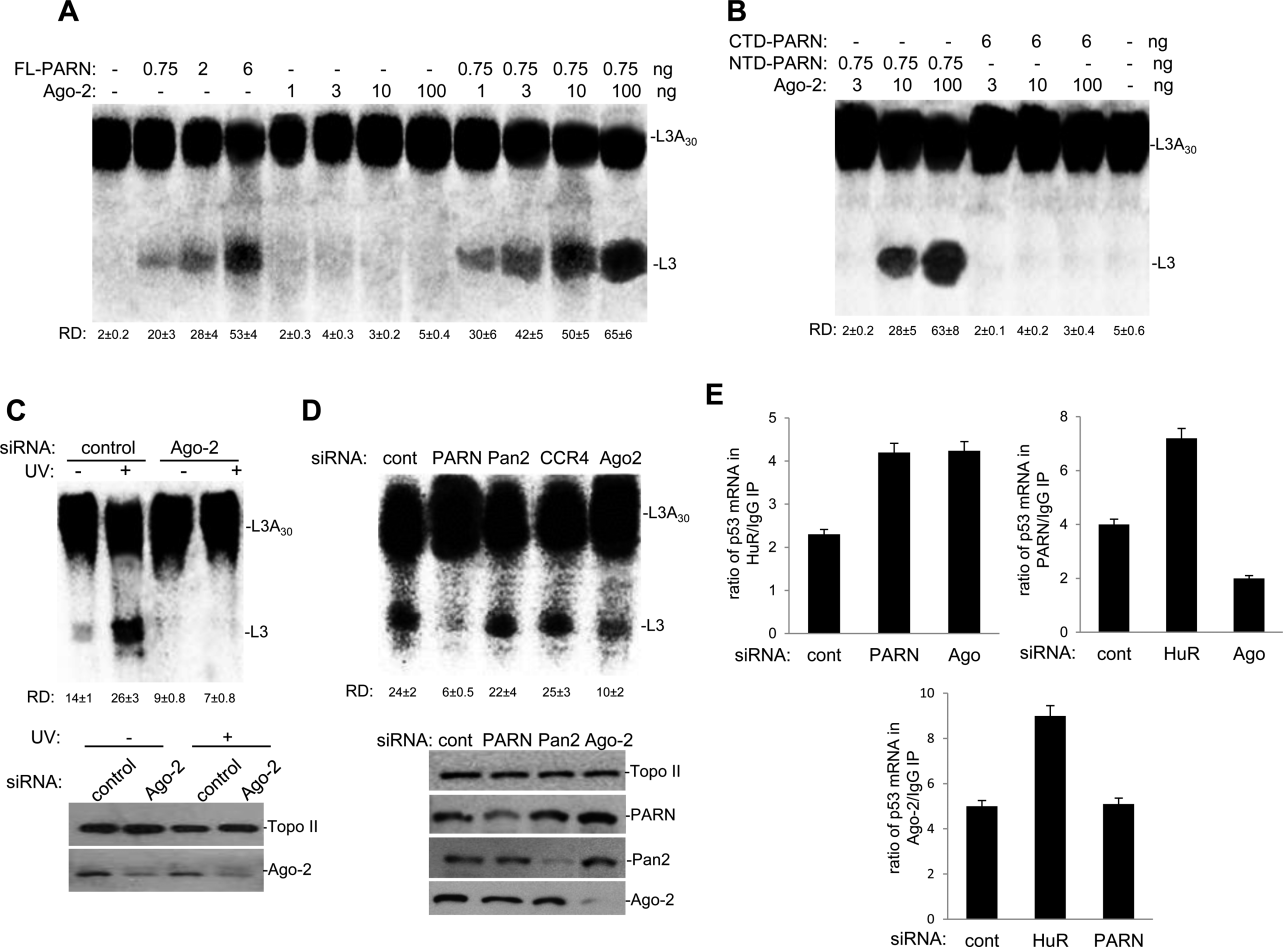
Ago-2 enhances PARN-mediated deadenylation. **(A)** Ago-2 can activate PARN-dependent deadenylation *in vitro*. Deadenylation assays were performed in the presence of radiolabeled capped L3(A_30_) RNA substrate using different concentrations of His-PARN and increasing amount of GST-Ago-2. Deadenylation reactions were performed for 90 min as described (4). Positions of the polyadenylated RNA L3(A_30_) and the L3 deadenylated product are indicated. Numbers beneath gel lane indicate relative deadenylation (RD), which was calculated as [L3 fragment/(L3(A_30_) + L3 fragment)] x 100. A representative deadenylation reaction from three independent assays is shown. The means ± standard deviation of RD values are indicated. Quantifications were done with Image J software. **(B)** Active PARN is necessary for Ago-2-mediated activation of deadenylation. Deadenylation assays were performed as in (A) with the addition of different His-PARN derivatives. The truncated forms of PARN used in this assay were described before (4) and include the C-terminal fragment of PARN (aa 443–639, His-CTD-PARN) and the N-terminal fragment of PARN (aa 1–470, His-NTD-PARN). Deadenylation reactions were analyzed as in (A). **(C)** siRNA-mediated knockdown of Ago-2 abolishes UV-induced activation of deadenylation in HCT116 cells. The protein levels of Ago-2 and Topo II were analyzed by western blotting after Ago-2/control siRNA treatment (Left). A representative deadenylation reaction from three independent assays is shown (Right). A representative western blot from three independent assays is shown. NEs from HCT116 cells treated with Ago-2/control siRNA and UV irradiation, and allowed to recover for 2 h were analyzed for radiolabeled L3(A_30_) deadenylation as described (4). RNAs were extracted and deadenylation reactions were analyzed as in (A). **(D)** Inhibition of nuclear deadenylation by Ago-2 and PARN knockdown but not by Pan2 and CCR4-NOT deadenylases knockdown. NEs from HCT116 cells treated with control, PARN, CCR4-NOT, Pan2 or Ago-2 siRNAs were analyzed for radiolabeled L3(A_30_) deadenylation. As antibodies against CCR4-NOT are not commercially available, we confirmed the knockdown of CCR4 by qRT-PCR (Supplementary Figure S1C). NEs were prepared and deadenylation reactions were analyzed as in (A). A representative western blot from three independent assays is shown. **(E)** HuR competes with PARN-associated Ago-2 for binding TP53 mRNA. Extracts from cells treated with the indicated siRNAs were IPed with the indicated antibodies. The endogenous nuclear RNA-IPed with the antibodies was quantified by qRT-PCR using primers specific for p53 mRNA. The qRT-PCR values were calculated from three independent samples. Errors represent the SD (*n* = 3).

To test whether Ago-2 has a direct influence on PARN deadenylase activity we performed *in vitro* reconstituted deadenylation assays, where we monitored the deadenylation of a radiolabeled L3(A_30_) RNA substrate in a reaction using limiting amounts of His-PARN and increasing amounts of GST-Ago-2. Addition of GST-Ago-2 enhanced the deadenylation activity of full-length PARN up to 2.4 folds (Figure 4A), indicating that Ago-2 is an activator of PARN activity in a cell-free system and in the absence of miRNA. The addition of GST-Ago2 to the N-terminal domain of PARN derivative, which contains the nuclease activity and binds Ago-2, increased deadenylation activity to similar extent that full-length PARN (Figure 4B). Importantly, we did not detect deadenylation activity when using GST-Ago-2 alone, GST-Ago-2 with a deadenylase-deficient PARN fragment (CTD-PARN derivative) or GST (Supplementary Figure S3B). Protein levels used in this assay are shown in Supplementary Figure S2. Consistent with our previous studies (4), ML43(G_15_) RNA substrate was unaffected by addition of recombinant PARN or a mix of recombinant PARN and Ago-2 to the deadenylation reaction (Supplementary Figure S3A), suggesting that the observed activity of the PARN/Ago-2 complex was a 3′ exonuclease and not endonucleolytic cleavage. Extending these results, we investigated the role of Ago-2 in deadenylation by performing siRNA mediated knockdown of Ago-2 in HCT116 cell line and then analyzing the UV-induced activation of deadenylation. As described in previous studies (4,5,53), Figure 4C shows that deadenylation activity in NEs of HCT116 cells treated with control siRNA increased significantly after UV treatment. Interestingly, deadenylation, especially after UV-treatment, was completely abolished in samples from cells treated with siRNA targeting Ago-2. While the knockdown of either PARN or Ago-2 resulted in inhibition of deadenylation using NEs; knockdowns of CCR4 or Pan2, which are involved in miRNA-mediated deadenylation in cytoplasmic fractions, did not have a significant effect on deadenylation in nuclear samples (Figure 4D). Our results indicate that PARN-mediated deadenylation is activated in the presence of Ago-2 both *in vitro* and in NEs from HCT116 cells. Together these results suggest that Ago-2/PARN complex formation activates PARN deadenylase and, therefore, might regulate gene expression.

Then we determined the effect of the expression of a well-known ARE-BP, HuR, on the binding of PARN and Ago-2 to TP53 mRNA. As HuR binds the U-rich element (encompassing positions 2181 to 2223) adjacent to miR-125b/miR-504 binding site (2,29–31), it is possible that it might affect the binding of PARN/Ago-2 to that region of TP53 3′ UTR. RNA-IP assays were performed using HCT116 cells treated with control, HuR, PARN or Ago-2 siRNAs and antibodies against either HuR, PARN, Ago-2 and IgG (Figure 4E). Either Ago-2 or PARN knockdown increased the association of HuR with the TP53 mRNA in samples from HCT116 cells. In the absence of HuR, the association of both PARN and Ago-2 with TP53 mRNA was favored. Consistent with the results shown in Figure 1E, Ago-2 knockdown caused a decrease in PARN-TP53 mRNA complexes. Cellular levels of PARN did not affect complex formation between Ago-2 and TP53 mRNA, supporting the idea that PARN does not bind directly TP53 mRNA and that Ago-2 facilitates PARN recruitment to TP53 3′ UTR. These results indicate that HuR can revert the binding of PARN-associated Ago-2 to TP53 3′ UTR. Consistent with this, the functional knockdown of miR-125b not only resulted in an increase of HuR protein levels (Figure 2C) but also in elongation of the poly(A) tail length of TP53 mRNA (Figure 2D), a decrease in the binding of PARN to TP53 mRNA (Figure 2E); and a decrease in the deadenylation of WT TP53 3′ UTR (Figure 2G). Interestingly, while HuR binds to TP53 3′ UTR in a UV-dependent manner enhancing TP53 mRNA translation (2), PARN-dissociates TP53 mRNA after UV treatment resulting also in an increase in p53 expression levels (5). While further studies are necessary to test the details of this mechanism, our data indicate that HuR can compete with PARN-associated Ago-2 for binding to TP53 3′ UTR, providing a mechanistic explanation for the changes in p53 expression during the progression of DDR.

## DISCUSSION

Deadenylation plays a crucial role in the control of mRNAs steady state levels and, hence, gene expression in different cellular conditions. Deadenylation is controlled by ARE and miRNA targeting sites within the 3′ UTR of eukaryotic mRNAs. Although numerous studies have shown that CAF1/CCR4/NOT1 and Pan2–Pan3 are complexes involved in miRNA-mediated deadenylation (reviewed in (1,18,19,35), the results presented here are the first to show that PARN also plays a role in this miRNA-dependent pathway in mammalian cells. As we previously showed that PARN can regulate the steady-state levels of TP53 mRNA under non-stress conditions (5), we used TP53 mRNA to test the functional interplay of RNA-BPs, mRNA target sequences, miRISC elements and PARN deadenylase during DDR. Our studies indicate that PARN deadenylase regulates TP53 mRNA stability and, consequently, expression levels through not only an ARE but also an adjacent miRNA-targeting site in the 3′ UTR. (Figure 1). Furthermore, our results indicate that components of mammalian miRISC complex, such as Ago-2 (Figure 1) and miR-125b (Figure 2), facilitate the binding of PARN to the target TP53 mRNA 3′ UTR resulting in TP53 mRNA poly(A) tail shortening and decrease in TP53 transcript and protein levels (Figure 2). PARN physically interacts with Ago-2 (Figure 3) resulting in the activation of PARN deadenylase activity (Figure 4). The ARE-BP HuR, which binds and stabilizes TP53 mRNA upon UV-treatment, competes with PARN and Ago-2 for binding to TP53 mRNA (Figure 4). Our studies with different cellular fractions (Figure 1G) and localization assays (Figure 3C and D), suggest that PARN-mediated deadenylation TP53 mRNA might be a phenomenon that can occur in both nuclear and cytoplasmic compartments. Taken together, our results provide new insights into the mechanism behind PARN-mediated regulation of deadenylation and gene expression, showing that PARN also plays a role in miRNA-dependent pathway when the seed sequences are adjacent/overlap an ARE in mammalian cells.

**Figure 5.**
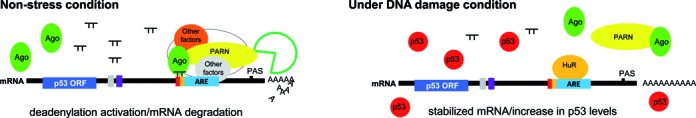
Model of multicomponent complexes required for regulation of TP53 mRNA steady state levels by PARN deadenylase in different cellular conditions. Cooperation of ARE-BPs, miRNAs, miRISC, PARN deadenylase and exosome is essential for the regulation of p53 mRNA stability in different cellular conditions. The recruitment of both ARE-BPs and miR-125b-loaded miRISC complexes to the ARE sequence and adjacent miR-125b targeting site, respectively, assist in the recruitment of PARN deadenylase to the target mRNA. Changes in the ARE-BPs bound to the 3′ UTR and miRNA abundance might signal the DDR. miRNA targeting sites present in p53 mRNA 3′ UTR are shown in different colors (miR-25 gray, miR-30d purple, miR-125b red, miR-504 orange).

Based on the results presented here, we propose a model in which, under non-stress conditions, PARN physically associates with TP53 mRNA through ARE and adjacent miRNA targeting sites in the TP53 by 3′ UTR. In those conditions, miR-125b-loaded miRISC, Ago-2 and ARE-BPs recruit PARN deadenylase to the target mRNA, resulting in the activation of deadenylation and low levels of p53 expression (Figure 5). After UV treatment, p53 expression levels increase and this occurs in correlation with a decrease in miR-125b levels (Figure 2A). Consistent with this, previous studies have shown that miR-125b is down-regulated upon χ-irradiation or camptothecin treatment, which leads to accumulation of p53 and apoptosis upon stress (31,54). As miR-125b plays a role in PARN/Ago-2 recruitment to the TP53 mRNA (Figure 2B–D), the UV-induced change in its content might explain, in part, the increase in p53 expression levels during DDR (Figure 5). Importantly, Ago-2 binds to a specific group of miRNAs under non-stress conditions (55), suggesting that changes in miRNA abundance might affect PARN/Ago-2 functions under different cellular conditions. It is important to highlight that our results indicate that PARN form a complex other miRNAs, such as miR-30d, in UV-dependent manner (Figure 2B). However, miRNA targeting sequences that were not adjacent to the ARE, such as the one for miR-30d, were not able to recruit PARN deadenylase to TP53 3′ UTR (Figure 1). Interestingly, our preliminary search indicated that two PARN target genes have a miR-30d-targeting site adjacent to ARE sequence in their 3′ UTRs. Those genes are CTGF (45,46) and TM4SF1 (47,48). Further studies are necessary to determine whether the PARN-miR-30d complex targets other mRNAs than TP53, and the relevance of miR-30d-targeting site overlapping an ARE sequence on PARN regulatory functions. Together these results indicate that PARN plays a role in miRNA-dependent pathway when a miRNA targeting site is located adjacent to an ARE element, and that Ago-2/PARN function might be dynamically controlled, partly, by miRNA levels under stress conditions.

The UV-induced changes in the binding of different ARE-BPs to the 3′ UTRs of the PARN target mRNAs might also influence the resulting expression levels during DDR (Figure 5). For example, it has been shown that after UV treatment ARE-BP HuR binds to AREs, resulting in the dissociation of tristetraprolin (TTP) and KH-type splicing regulatory protein (KSRP) from ARE-containing mRNAs and the up-regulation of genes involved in DDR (53). In fact, HuR binds to TP53 mRNA in a UV-dependent manner (2). HuR binds its substrates in the nucleus, as early as co-transcriptionally, and escort them to the cytoplasm where translation of TP53 mRNA is increased (56,57). In a recent study, it has been reported that over 75% of mRNAs with Ago binding sites in the 3′ UTR also have HuR binding sites (57). Most of these Ago and HuR binding sites overlap or are adjacent, with a distance of less than 10 nt of one another, suggesting a potential competitive or cooperative regulation of target mRNAs (57). Besides, HuR sites in 3′ UTRs of mRNAs overlap extensively with predicted miRNA target sites (58), suggesting interplay between the functions of HuR and miRNAs. Consistent with those studies, our results indicate that not only ARE sequences, which are involved in the binding of TTP, KSRP or HuR, but also an overlapping/adjacent miRNA targeting site are involved in the functional recruitment of PARN to TP53 mRNA. Interestingly, 20% of PARN mRNA targets in the p53 signaling pathway (5) showed physical overlapping of an ARE and miRNA target site in their 3′ UTRs (Supplementary Figure S4). Further studies would reveal the role of other ARE-BPs in this PARN-associated regulatory pathway, the identity of other mRNA targets and whether the proximity of these sequence elements is important in this pathway.

While the exact contribution of miRNAs, miRISC, AREs and ARE-BPs to mRNA decay has not been elucidated yet, recent studies have described a functional overlap between ARE- and miRNA-mediated mRNA turnover pathways (1). Although some studies have shown that ARE-mediated decay can occur independent of miRNA functions (14), an increasing number of publications have shown that miRNAs can functionally interact with ARE-BPs, and that Dicer and Ago are required for ARE-mediated decay. For example, it has been shown that HuR can bind to AREs present in c-myc 3′ UTR at a site proximal to that recognized by let-7 miRNA, facilitating the targeting of let-7-loaded miRISC and mediating the reduction of c-Myc mRNA levels (38). Furthermore, ARE-BP Nucleolin is necessary for miR-130a and mir-301a mediated deadenylation of CSF-1 mRNA (59). Another example is the functional interaction of ARE-BP TTP and miRISC that results in the recruitment of the deadenylase and the exosome for tumor necrosis factor-α mRNA degradation (37).

Although the miRISC has been shown to interact with several deadenylases resulting in the regulation of mRNA stability and gene expression (reviewed in (19), the results presented here are the first to show a functional interaction with PARN. Recently, it has been shown that PARN and one of the elements of the miRISC, Ago-2, functionally interact playing a role in miRNA biogenesis pathway (36). However, no details on the PARN-Ago-2 interaction have been provided in that study. Taken together, our studies provide evidence of the functional interaction of PARN with elements of the ARE- and miRNA-regulatory pathways when both signals are adjacent or overlap in the 3′ UTR of target genes. Our studies also provide new insights into the mechanisms behind the regulation of p53 expression.

## Supplementary Material

SUPPLEMENTARY DATA
