# Supplementary material for: PARN deadenylase is involved in miRNA-dependent degradation of TP53 mRNA in mammalian cells
Source: Nucleic Acids Res. 2015 Sep 22;43(22):10925–38. doi: 10.1093/nar/gkv959 (PMC4678859; doi:10.1093/nar/gkv959)
Supplement: SUPPLEMENTARY DATA [file supp_43_22_10925__index.html]

PARN deadenylase is involved in miRNA-dependent degradation of TP53 mRNA in mammalian cells — PARN deadenylase is involved in miRNA-dependent degradation of TP53 mRNA in mammalian cells — SUPPLEMENTARY DATA 

# PARN deadenylase is involved in miRNA-dependent degradation of TP53 mRNA in mammalian cells

## SUPPLEMENTARY DATA

- SUPPLEMENTARY DATA
