# Supplementary material for: PARN deadenylase is involved in miRNA-dependent degradation of TP53 mRNA in mammalian cells
Source: Nucleic Acids Res. 2015 Sep 22;43(22):10925–38. doi: 10.1093/nar/gkv959 (PMC4678859; doi:10.1093/nar/gkv959)
Supplement: SUPPLEMENTARY DATA [file supp_gkv959_nar-01074-a-2015-File007.pdf]

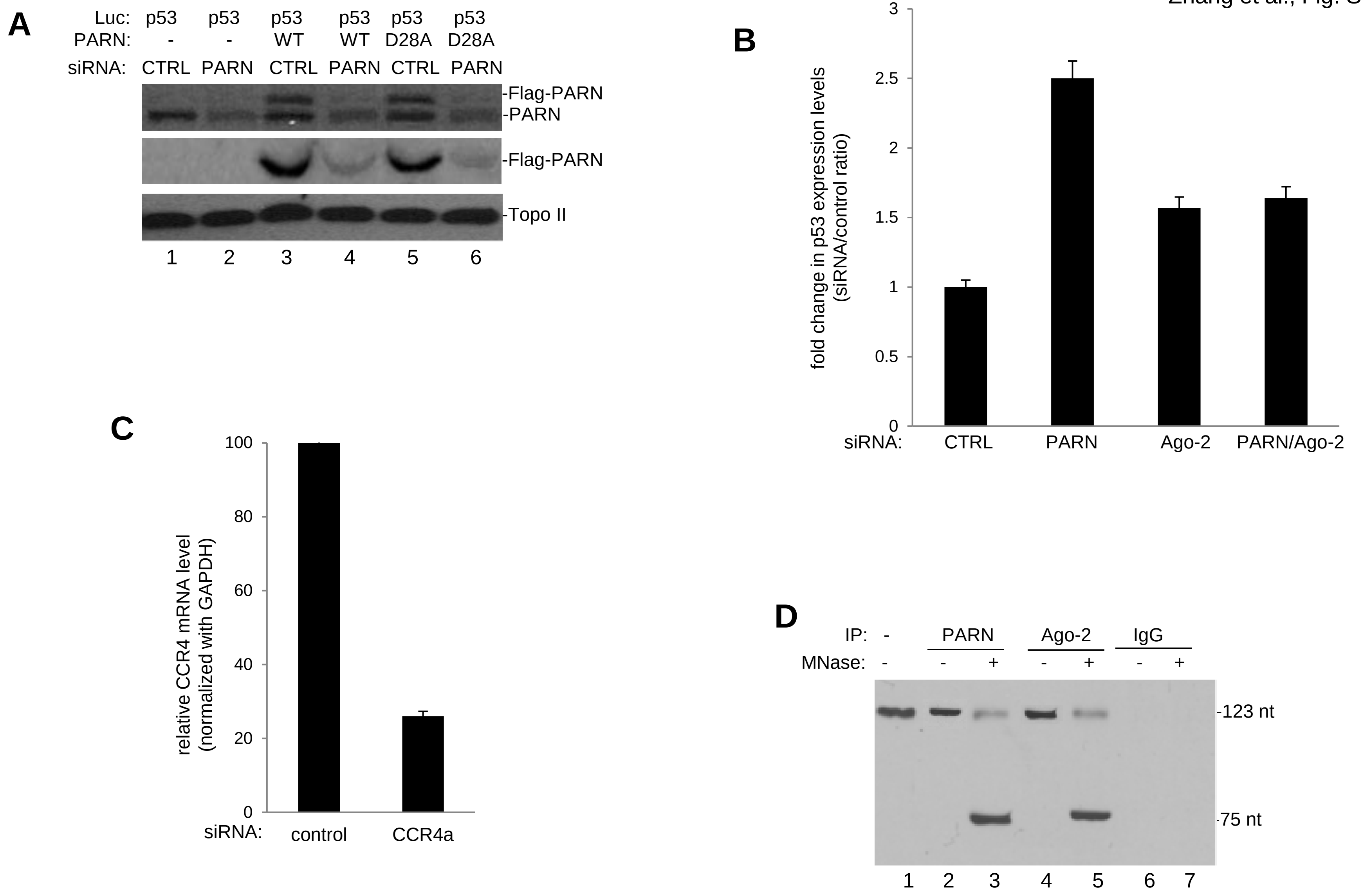
A
Luc: p53 p53 p53 p53 p53 p53
PARN: - - WT WT D28A D28A
siRNA: CTRL PARN CTRL PARN CTRL PARN
-Flag-PARN
-PARN
-Flag-PARN
-Topo II
1 2 3 4 5 6
B
fold change in p53 expression levels (siRNA/control ratio)
0 0.5 1 1.5 2 2.5 3
siRNA: CTRL PARN Ago-2 PARN/Ago-2
C
relative CCR4 mRNA level (normalized with GAPDH)
0 20 40 60 80 100
siRNA: control CCR4a
D
IP: - PARN Ago-2 IgG
MNase: - - + - + - +
-123 nt
-75 nt
1 2 3 4 5 6 7

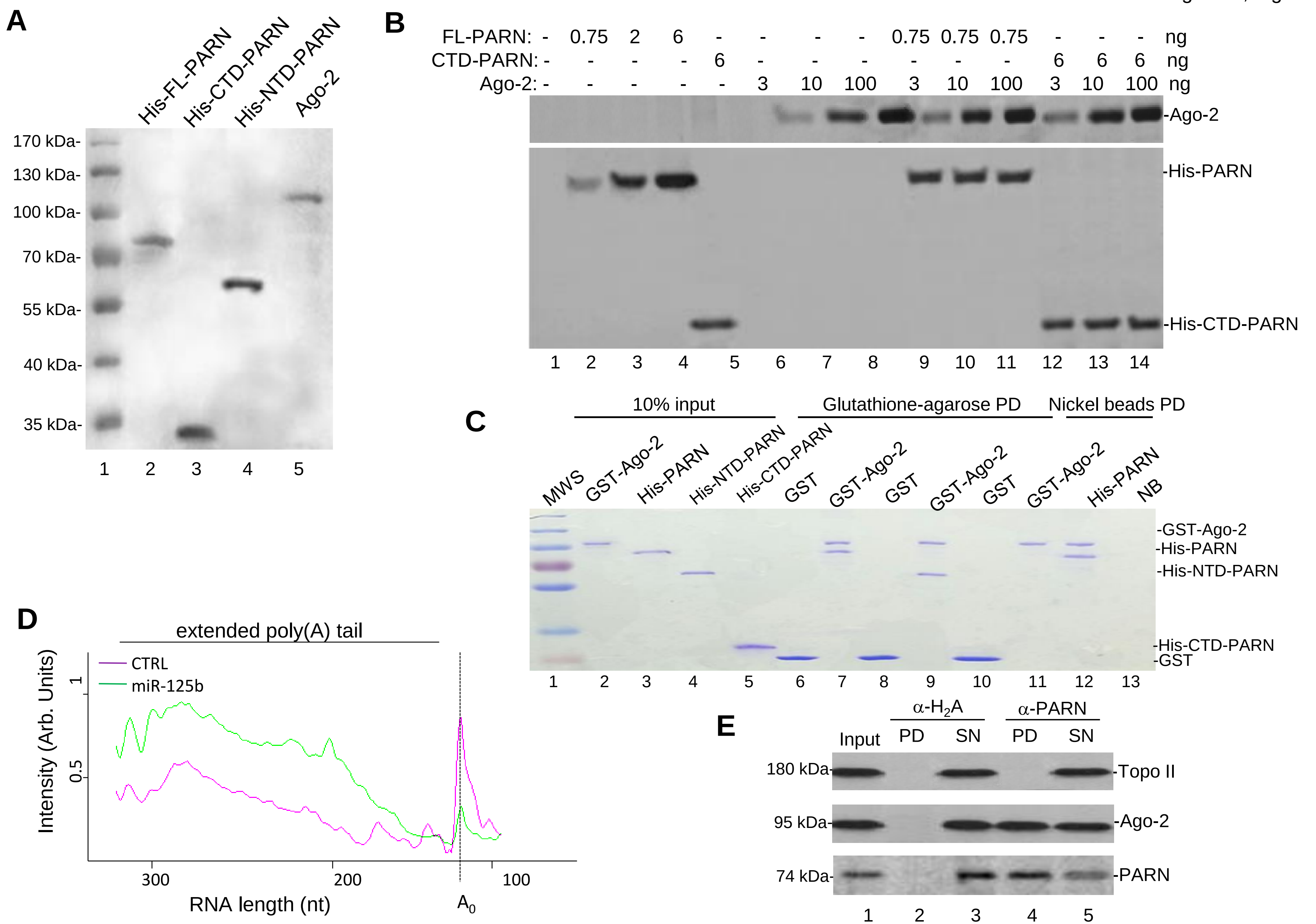
A
His-FL-PARN
His-CTD-PARN
His-NTD-PARN
Ago-2
170 kDa-
130 kDa-
100 kDa-
70 kDa-
55 kDa-
40 kDa-
35 kDa-
1 2 3 4 5
B
FL-PARN: - 0.75 2 6 - - - - 0.75 0.75 0.75 - - - ng
CTD-PARN: - - - - 6 - - - - - - 6 6 6 ng
Ago-2: - - - - - 3 10 100 3 10 100 3 10 100 ng
-Ago-2
-His-PARN
-His-CTD-PARN
1 2 3 4 5 6 7 8 9 10 11 12 13 14
C
10% input
Glutathione-agarose PD
Nickel beads PD
MWS
GST-Ago-2
His-PARN
His-NTD-PARN
His-CTD-PARN
GST
GST-Ago-2
GST
GST-Ago-2
GST
GST-Ago-2
His-PARN
NB
-GST-Ago-2
-His-PARN
-His-NTD-PARN
-His-CTD-PARN
-GST
1 2 3 4 5 6 7 8 9 10 11 12 13
D
extended poly(A) tail
CTRL
miR-125b
Intensity (Arb. Units)
1
0.5
300
200
100
RNA length (nt)
A0
E
α-H2A
α-PARN
Input
PD
SN
PD
SN
180 kDa-
95 kDa-
74 kDa-
-Topo II
-Ago-2
-PARN
1 2 3 4 5

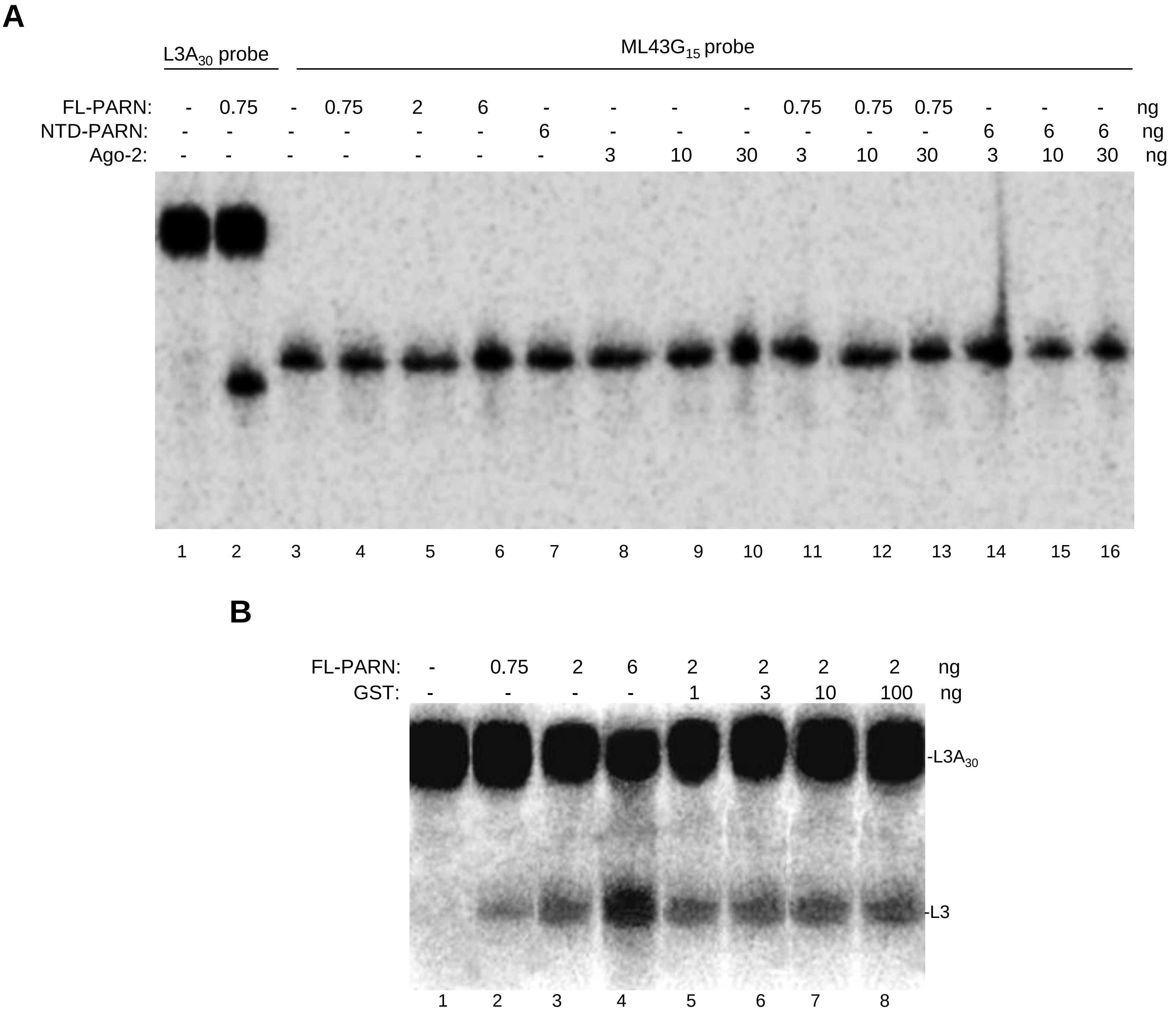
A
L3A30 probe
ML43G15 probe
FL-PARN: - 0.75 - 0.75 2 6 - - - - 0.75 0.75 0.75 - - - ng
NTD-PARN: - - - - - - 6 - - - - - - 6 6 6 ng
Ago-2: - - - - - - - 3 10 30 3 10 30 3 10 30 ng
1 2 3 4 5 6 7 8 9 10 11 12 13 14 15 16
B
FL-PARN: - 0.75 2 6 2 2 2 2 ng
GST: - - - - 1 3 10 100 ng
-L3A30
-L3
1 2 3 4 5 6 7 8

Zhang et al., Fig. S4

Gene Symbol	Description	miRNA targeting sites at the 3'UTR	ARE binding proteins
TP53	tumor protein p53	miR125b, miR504, miR25, miR-30d	Wig-1, HuR
CHEK2	CHK2 checkpoint homolog (S. pombe)	miR-24-2	unknown
CDK2	cyclin-dependent kinase 2	miR-372, miR-205, miR-885-5p, miR-103, miR-16	unknown
ANXA1	annexin A1	miR-21, miR-584, miR-196a and miR-196b	unknown
PEG10	paternally expressed 10	miR-122	unknown
ID1	inhibitor of DNA binding 1	miR-206	unknown
FOS	FBJ murine osteosarcoma viral oncogene	miR-29b, miR-155, miR-101, miR-221/222, miR-7b,	HuR, AUF1, TTP
V-MYC	v-myc myelocytomatosis viral oncogene	miR-26a, miR-24, miR-378, let-7, miR-17-92, miR-145, etc.	HuR, AUF1, TTP
PTEN	phosphatase and tensin homolog	miR-21, miR-17, miR-20a, miR-106b, miR-221, etc.	TTP
RBL2	retinoblastoma-like 2 (p130)	miR302/367, miR-17, miR-20a, miR-106b, miR-290, etc.	unknown
MYO6	myosin VI	miR-143 and miR-145	unknown
CD47	CD47 molecule	miR-34a, miR-155 and miR-326	unknown
RB1CC1	RB1-inducible coiled-coil 1	miR-138	unknown
MBNL2	muscleblind-like 2 (Drosophila)	miR-302d, miR-372, miR-200c	unknown
BCL2L1	BCL2-like 1	miR-21	unknown
MMP1	matrix metallopeptidase 1	miR-222	unknown
THBS1	thrombospondin 1	miR-17-92	HuR
LIF	leukemia inhibitory factor	miR-346, miR-494, miR-199	Unknown
SMAD3	SMAD family member 3	miR-135a, miR-140-5p, miR-582-3p, miR-582-5p, etc.	Unknown
IL8	interleukin 8	miR-146a	unknown
PLAU	plasminogen activator, urokinase	miR-193a	unknown
FN1	fibronectin 1	miR-200c, miR-29b	unknown
CCND3	cyclin D3	miR-16	unknown
SERPINE1	serpin peptidase inhibitor, clade E (type 1)	miR-146a, miRNA-449a/b, miR-30c and miR-301a	HuR
HDAC1	histone deacetylase 1	miR-449a	unknown
CTGF	connective tissue growth factor	miR-17-92, miR-18, miR-19, miR-133 and miR-30	HuR
CCND1	cyclin D1	miR-15a, miR-16, miR-302, miR-205, let-7, miR-34a, etc.	TTP, HuR, KSRP
VEGFA	vascular endothelial growth factor A	miR-297, miR-299, miR-567, miR-609, miR-222, etc.	HuR

SUPPORTING INFORMATION

Figure S1. **A)** The expression levels of both WT and catalytically inactive PARN was similar. Constructs with either WT PARN or catalytically inactive D28A PARN were co-transfected with firefly luciferase reporter constructs with 3'UTR sequences from the TP53 gene (Figure 1B) into HCT116 cells treated with control or PARN siRNA. Equivalent amounts of nuclear extracts were resolved by SDS-PAGE and proteins were detected by immunoblotting using antibodies against PARN and Flag. Antibody against Topo II was used as a control. A representative Western blot from three independent samples is shown. **B)** siRNA-mediated knockdown of Ago-2 and PARN have similar effects on p53 expression levels. NEs from HCT116 cells treated with control, PARN, Ago-2, or both PARN/Ago-2 siRNAs were analyzed by Western blot for p53 expression. Topo II was used as loading control. The p53 protein expression levels were calculated from three independent samples. The basal level of the proteins was arbitrarily set at 1.0 in the control siRNA-treated sample. Errors represent the SD derived from three independent experiments. **C)** siRNA-mediated knockdown of CCR4. As antibodies against CCR4-NOT are not commercially available, we confirmed the knockdown of CCR4 by qRT-PCR. Errors represent the SD derived from three independent experiments. **D)** PARN and Ago-2 bind the same region of TP53 3'UTR as part of the same complex. RPA assays were performed using either PARN or Ago-2 antibodies for RIP; NEs from HCT116 cells; and a radiolabeled RNA encompassing the ARE adjacent to the miR-504/miR-125b-targeting site of the TP53 3'UTR (123 nt). Samples were crosslinked before RIP. After RIP, samples were treated with MNase and proteins were eliminated by proteinase K treatment, phenol/chloroform extraction and precipitation. Samples were analyzed in denaturing urea gels followed by autoradiography. A representative RPA reaction from three independent assays is shown.

Figure S2. **A)** Recombinant proteins used in pull-down (Figure 3B) and *in vitro* deadenylation (Figures 4A-B) assays. Coomassie blue staining of the purified recombinant proteins after SDS–PAGE is shown. **B)** Concentrations of recombinant proteins used in *in vitro* deadenylation (Figures 4A-B) are shown. Proteins were resolved by SDS–PAGE and detected with anti-PARN or anti-Ago-2 antibodies. **C)** Ago-2 interacts directly with the N-terminal domain of PARN. Immobilized GST-Ago-2 or GST on glutathione beads were incubated with full-length (His-PARN), N-terminal domain (His-NTD-PARN; aa 1–470) or C-terminal domain (His-CTD-PARN; aa 443– 639). Immobilized His-PARN on nickel beads or nickel beads alones (NB) were also incubated with GST-Ago-2. Bound proteins were eluted, and analyzed by Coomassie blue staining. 10% of either His-PARN derivatives or GST-Ago-2 used in the reactions are shown as input. Molecular weight standard (MWS) was included. A representative pull-down reaction from three independent assays is shown. **D)** Quantification of poly(A) tail length in Figure 2D was done by obtaining the density profile of control and miR-125b inhibitor treated lanes using Image J software. **E)** Control of specificity for the IPs shown in Figure 3A. NEs were IPed with either anti-PARN or anti-H2A antibodies. As preimmune antibodies were not available, we used a non-specific antibody, anti-H2A, as in a previous publication (4). Equivalent amounts of the pellets (IP) and supernatants (SN) were resolved by SDS-PAGE and proteins were detected by immunoblotting using antibodies against PARN and Ago-2. Antibody against Topo II was used as a control. A representative pull-down reaction from three independent assays is shown.

Figure S3. *In vitro* deadenylation assays shown in Figure 4A-B were performed in the presence of L3(A_{30}) and ML43(G_{15}) RNA substrates radioactively labelled in their RNA body. No endonucleolytic cleavage activity was detected when recombinant PARN and/or Ago-2 proteins were incubated with ML43(G_{15}) substrate. The reactions were analyzed by electrophoresis on 10% polyacrylamide/7M urea gels. **B)** Control of specificity for deadenylation reactions shown in Figures A-B. Concentrations of GST protein similar to those used in the reactions shown in Figure 4A-B were in added. Deadenylation assays were performed in the presence of radiolabeled capped L3(A_{30}) RNA substrate using different concentrations of His-PARN and increasing amount of GST. Deadenylation reactions were performed for 90 min as described (4). Positions of the polyadenylated RNA L3(A_{30}) and the L3 deadenylated product are indicated. A representative deadenylation reaction from three independent assays is shown.

Figure S4. Analysis for the presence of AREs and miRNA targeting sites in the 3'UTRs of genes from the p53 pathway affected by PARN expression in non-stress conditions. The expression of 141 genes from the p53 pathway was affected upon siRNA-mediated knockdown of PARN (5). 20% of these genes, including TP53, showed both target sites. ARED 3.0 database and miRWalk were used for the search of ARE-containing mRNAs and miRNAs validated targets (58), respectively.
